# The Response of Macrophages in Sepsis-Induced Acute Kidney Injury

**DOI:** 10.3390/jcm12031101

**Published:** 2023-01-31

**Authors:** Jiawei He, Shen Zhao, Meili Duan

**Affiliations:** Department of Critical Care Medicine, Beijing Friendship Hospital, Capital Medical University, Beijing 100050, China

**Keywords:** sepsis, acute kidney injury, macrophages, phenotype, inflammation

## Abstract

Sepsis-induced acute kidney injury (SAKI) is common in critically ill patients and often leads to poor prognosis. At present, the pathogenesis of SAKI has not been fully clarified, and there is no effective treatment. Macrophages are immune cells that play an important role in the pathogenesis of SAKI. The phenotype and role of macrophages can vary from early to later stages of SAKI. Elucidating the role of macrophages in SAKI will be beneficial to its diagnosis and treatment. This article reviews past studies describing the role of macrophages in SAKI, with the aim of identifying novel therapeutic targets.

## 1. Introduction

Sepsis is a dysregulated immune response caused by an infection that can lead to multiple-organ dysfunction. Sepsis-induced acute kidney injury (SAKI) is a common complication of sepsis that can result in increased mortality and poor prognosis [1]. Due to a lack of understanding of the pathogenesis of SAKI, no effective treatments currently exist. While waiting for renal function to recover, we can use renal replacement therapy to maintain volume balance and homeostasis. However, this does not reduce the incidence of SAKI and the risk of progression to chronic kidney disease [2]. Therefore, elucidating the pathogenesis of SAKI will help to raise awareness of the disease, which may lead to the discovery of new therapeutic targets. The primary pathogenesis of SAKI is an immune imbalance, which is characterized by an imbalance between pro-inflammatory and anti-inflammatory responses whereby the stronger pro-inflammatory response results in tissue cell damage, while the function of anti-inflammatory immune cells is impaired. Although inhibition of inflammation can reduce the severity of SAKI, it can also increase the risk of infection [3]. Understanding immune balance in SAKI will shed light on both its pathogenesis and potential treatments.

Macrophages are immune cells that play an important role in the homeostasis of tissues and organs. In the heart, macrophages are necessary for normal electrical conduction [4]. In the intestine, they interact with neurons to promote peristalsis [5]. More and more studies have also focused on renal macrophages, which may regulate the processes of renal infection, ischemia reperfusion injury, drug toxicity, diabetic nephropathy, and other diseases [6]. Overall evidence shows that renal macrophages contribute to the renal injury response both positively and negatively [7]. This article will focus on the different roles of renal macrophages across the phases of SAKI disease progression, in order to identify new targets for SAKI treatment.

## 2. Macrophage Origins

**Figure 1:**
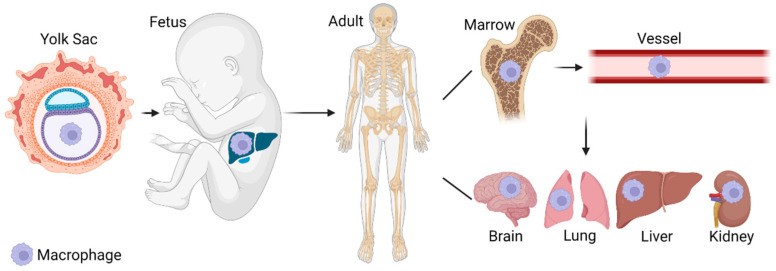
Source of macrophages. Macrophages are originally derived from the yolk sac, mainly from the liver during the embryonic period, and are mainly stored in the bone marrow after delivery. Hematopoietic stem cells in the bone marrow differentiate into macrophages under the action of cytokines and then enter the blood circulation to all parts of the body. In addition to circulating macrophages in the blood, various tissues and organs of the body also contain resident macrophages. For example, microglia in the brain, alveolar macrophages in the lung, Kupffer cells in the liver, and macrophages in the kidney, etc.

Macrophages differentiate from various tissues throughout organism development. In the embryonic stage, macrophages are mainly derived from the yolk sac. In the fetal period, macrophages are mainly derived from the liver. After delivery, macrophages are mainly derived from bone marrow [8]. Although macrophages were initially thought to be derived from hematopoietic stem cells in the bone marrow through monocyte differentiation [9], recent studies in mice have confirmed that some tissue macrophages are inoculated prenatally and therefore originate from yolk sac or fetal liver progenitor cells. Compared with macrophages transformed from monocytes in the blood, renal macrophages play a more important role in maintaining the homeostasis of tissues and organs (Figure 1) [10]. Kidneys from newborn mice contain a prominent population of embryonic-derived MHCII^neg^F4/80^hi^CD11b^low^ macrophages that express mucin domain containing 4 (TIM-4) and MER receptor tyrosine kinase (MERTK). These macrophages are replaced within a few weeks after birth by phenotypically similar cells that express MHCII but lack TIM-4 and MERTK [11]. Resident macrophage change also occurs associated with AKI. Macrophages with an embryonic phenotype re-appear with transcriptional reprogramming, more closely resembling resident macrophages at birth including enrichment of transcripts related to Wnt signaling [12]. This suggests that macrophage mechanisms involved in kidney development may be contributing to kidney repair after injury.

## 3. Macrophage Subtypes

Previous studies have divided renal macrophages into pro-inflammatory M1 and anti-inflammatory M2 types, a classification system that has been beneficial to many preclinical studies [13]. But recently, more and more researchers have found that macrophages cannot be so simply divided. M1 and M2 are typical examples of macrophage polarization, but there are a series of transitional macrophages between M1 and M2 that also play distinct roles [14]. Kawakami et al. studied mouse kidney macrophages and found that renal macrophages can be divided into five categories according to the markers on the cell surface [15]. Their five macrophage types also showed different functions, such as secreting inflammatory factors or phagocytic antigens and activating T cells. Nordlohne et al. carried out additional research and found that cluster of differentiation 206 (CD206) was useful for further subdividing the five macrophage types. Interestingly, their results have been replicated in other animal models, such as ischemia reperfusion injury (IRI), unilateral ureteral ligation (UUO), and Alport syndrome. This implies that all subsets contribute in some way to inflammation and/or repair during inflammation [16]. There were significant differences between CD206+ and CD206- cells in terms of phagocytic ability and disease stages.

In addition to the classification of macrophage types within an organism, Zimmerman et al. noted differences in renal macrophages across species and genera [17]. For example, F4/80 is specific to mouse macrophages; the corresponding marker of macrophages in the human kidney is CD14 [18]. To identify a specific marker of cross-species renal macrophages, they extracted mouse, rat, pig, and human renal macrophages and performed single-cell RNA sequencing. This offered an unbiased analysis wherein cells with similar transcriptional groups were clustered without any preconceived ideas about their typical markers [19]. Using this technology, they identified the expression of CD74 and CD81 in a cross-species immune cell population of the kidney. Finally, a conjoined symbiosis experiment in mice showed that renal macrophages expressing CD74 and CD81 were not exchanged, confirming that these markers can be used to identify renal resident macrophages (Table 1).

## 4. Pro-Inflammation and Injury

Currently, it is believed that immune inflammatory response, microcirculation disturbance, and apoptosis are the combined causes of SAKI [20]. The immune complex activates Fc receptors on the surface of renal macrophages, leading to the recruitment of circulating monocytes into the kidney. In addition to their ability to recognize foreign substances and recruit inflammatory cells, renal macrophages can also produce inflammatory cytokines that cause damage to renal tubular epithelial cells [21]. The infection causes macrophages to produce pro-inflammatory cytokines such as interleukin-1β (IL-1β), IL-12, IL-18, tumor necrosis factor-α (TNF-α), and interferon-α/β (IFN-α/β), all of which lead to cell damage during SAKI. Pathogens activate macrophages through surface lipopolysaccharide (LPS), and can also be captured by natural killer cells through the release of IFN-γ. The major signaling pathways of pro-inflammatory cytokines produced by macrophages include Janus kinase (JAK)-signal transducer and activator of transcription (STAT) [22], nuclear factor κB (NF-κB) [23], interferon regulatory factor 3 (IRF3) [24], and phosphatidylinositol 3-kinase (PI3K)-protein kinase B (PKB, also known as AKT)-mammalian target of rapamycin (mTOR) pathway [25].

### 4.1. JAK-STAT

The JAK-STAT signaling pathway is a chain of interactions between proteins in a cell and is involved in processes such as immunity, cell division, cell death, and tumor formation. The pathway communicates information from chemical signals outside of a cell to the cell nucleus, resulting in the activation of genes through the process of transcription. There are three key parts of JAK-STAT signaling: JAK, STAT, and receptors which bind the chemical signals [26]. NK cells activated by pathogens can release IFN-γ, which can further activate macrophages by binding to the IFN-γ receptor on the surface of macrophages. JAK-STAT is the main signaling pathway for IFN-γ receptor activation, which enables macrophages to produce inflammatory cytokines through receptor interacting serine threonine protein kinase, nucleotide binding oligomerization domain like receptors family pyrin domain containing 3 (NLRP3) [27]. NLRP3 is a component of the innate immune system that functions as a pattern recognition receptor (PRR) that recognizes pathogen-associated molecular pattern (PAMP). NLRP3 belongs to the NOD-like receptor (NLR) subfamily of PRRs and NLRP3 together with the ASC forms a caspase-1 activating complex known as the NLRP3 inflammasome. NLRP3 in the absence of activating signal is kept in an inactive state in the cytoplasm. NLRP3 inflammasome detects danger signals and recruits ASC protein and caspase-1 to the inflammasome complex. Caspase-1 within the activated NLRP3 inflammasome complex in turn activates the inflammatory cytokine, IL-1β [28].

### 4.2. NF-κB

The nuclear factor kappa-light-chain-enhancer of activated B cells (NF-κB) is a protein complex that controls the transcription of DNA, cytokine production, and cell survival. LPS on the surface of pathogens is an important substance for the activation of macrophages. After being activated by LPS, TLR4 on the surface of macrophages can activate IKK-IκB through the MyD88-TAK1 pathway. With the degradation of IκB, the NF-κB complex is then freed to enter the nucleus where it can ‘turn on’ the expression of specific genes that have DNA-binding sites for NF-κB nearby [29]. The activation of these genes by NF-κB then leads to the given physiological response, for example, producing inflammatory cytokines such as IL12, IL18, and TNFα. Regulation of these signaling pathways can affect macrophage function: for example, inhibiting the phosphorylation of p65 in NF-κB can reduce the production of pro-inflammatory factors by macrophages [30]. Extracellular vesicles from neutrophils can also affect macrophages. Neutrophils produce extracellular vesicles that regulate NF-κB in macrophages, up-regulate the expression of NLRP3 in macrophages, and enhance the ability of macrophages to secrete pro-inflammatory factors [31].

### 4.3. IRF3-IFN

The activated TLR4 can also activate IRF3 via TRIF. IRF3 is a member of the interferon regulatory transcription factor (IRF) family. IRF3 is found in an inactive cytoplasmic form that upon serine/threonine phosphorylation forms a complex, which translocates into the nucleus for the transcriptional activation of interferons α/β, and further interferon-induced genes [32]. In addition, other studies have shown that IRF3 plays a role in identifying material damage. STING is a protein located in the endoplasmic reticulum of macrophages, capable of sensing double-stranded DNA in the cytoplasm and activating IRF3 through non-classical pathways. This may be one way that macrophages engulf and remove genetic material from pathogens [33].

### 4.4. PI3K-AKT-mTOR

**Figure 2:**
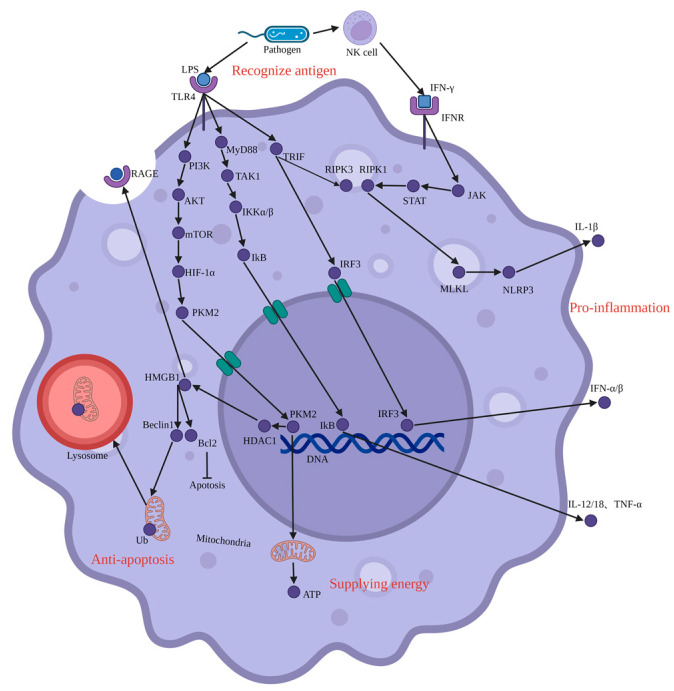
The signaling pathway of macrophages when releasing inflammatory factors. Macrophages recognize the lipopolysaccharide (LPS) of pathogens or the interferon (IFN)-γ released by natural killer (NK) cells. Macrophages produce interleukin (IL)-1β, IL-12, IL-18, tumor necrosis factor alpha (TNF-α), IFN-α/β and other pro-inflammatory factors through Janus kinase (JAK)-signal transducer of activators of transcription (STAT), toll-like receptors 4 (TLR4), nuclear factor kappa B (NF-κB), interferon regulatory factor 3 (IRF3) and other pathways. At the same time, macrophages also undergo metabolic reprogramming. The increase of pyruvate kinase isozymes M2 (PKM2) in macrophages, on the one hand, can induce mitochondria to produce ATP to supply energy, on the other hand, it can lead to the increase of high mobility group box 1 protein (HGBM1) through histone deacetylase 1 (HDAC1), through the phosphatidylinositol 3-kinase (PI3K)-protein kinase B (PKB, also known as AKT)-mammalian target of rapamycin (mTOR) pathway. HGBM1 can phagocytize LPS through the receptor of advanced glycation end products (RAGE) on the surface of macrophages and lead to the dissociation of Beclin1 and Bcl2. Beclin1 can induce autophagy of damaged mitochondria through phosphatase and tensin homologue deleted on chromosome (PTEN) induced putative kinase 1 (PINK1)-Parkin, and Bcl2 can avoid the death of macrophages. The common role of both is to maintain the survival of macrophages.

In addition to the release of pro-inflammatory factors, macrophages can also exert effects through their metabolic activity. After toll-like receptor 4 is activated by LPS, macrophages produce more pyruvate kinase muscle isozyme 2 (PKM2) through the PI3K-AKT-mTOR pathway [34]. PKM2 can induce the Warburg effect in mitochondria, and excessive lactic acid can regulate histone deacetylase 1 activity, resulting in the acetylation of high mobility group box 1 (HMGB1) [35]. Although excessive HMGB1 in circulation can make inflammation difficult to control, there are still many positive effects in the early stage of SAKI [36]. HMGB1 not only promotes macrophage uptake of LPS [37], but also inhibits macrophage apoptosis [38]. Although PKM2 can induce mitochondria to produce adenosine triphosphate in response to macrophage activation, it can also lead to mitochondrial mass decline [39]. When this occurs, Beclin1 promotes autophagy of damaged mitochondria through the PINK1-Parkin pathway. Most scholars believe that mitochondrial autophagy helps to improve the function of macrophages and inhibit macrophage apoptosis [40]. However, some have proposed that inhibition of mitochondrial autophagy can also enhance the activity of macrophages (Figure 2) [41].

## 5. Macrophage Transformation

When macrophages undergo metabolic reprogramming, their phenotypes change. Studies have shown that during SAKI, damaged renal tubular epithelial cells activate macrophages by releasing Sin3A associated protein 130 (SAP130) [42], aquaporin 1 (AQP1) [43], and chemokine C-X3-C motif ligand 1 (CX3CL1) [44], and promote the transformation of macrophages from pro-inflammatory to anti-inflammatory by affecting the genetic material of macrophages, such as through miR-219c-3p. SAP130 released from damaged renal tubule cells may induce macrophage activation and necrotic inflammation. This was demonstrated in vivo, where the application of SAP130 rich supernatant from dead tubular epithelial cells or recombinant SAP130 promoted Macrophage-inducible C-type lectin (Mincle) expression and macrophage accumulation, and severe renal tubulointerstitial inflammation of macrophages worsened in LPS induced Mincle wild type mice, but not in Mincle deficient mice. Further studies found that Mincle is negatively regulated by miR-219c-3p in macrophages because miR-219c-3p binds to Mincle 3’-UTR to inhibit Mincle translation [45]. At the same time, damaged renal tubular epithelial cells can also release extracellular vesicles containing genetic material (such as miR-19b-3p) that directly affects macrophage gene expression, thus inducing macrophages to change from pro-inflammatory to anti-inflammatory type. Global microRNA(miRNA) expression profiling of renal exosomes was examined in a SAKI mouse model and miR-19b-3p was identified as the miRNA that was most notably increased in tubular epithelial cells derived exosomes compared to controls. Similar results were also found in an adriamycin (ADR) induced chronic proteinuric kidney disease model in which exosomal miR-19b-3p was markedly released. Interestingly, once released, tubular epithelial cells derived exosomal miR-19b-3p were internalized by macrophages, leading to M1 phenotype polarization through targeting NF-κB. Importantly, the pathogenic role of exosomal miR-19b-3p in initiating renal inflammation was revealed by the ability of adoptively transferred purified tubular epithelial cells derived exosomes to cause tubulointerstitial inflammation in mice, which was reversed by inhibition of miR-19b-3p. Clinically, high levels of miR-19b-3p were found in urinary exosomes and were correlated with the severity of tubulointerstitial inflammation in patients with diabetic nephropathy. Thus, this study suggests that damaged renal tubular epithelial cells may promote phenotype changes in macrophages by releasing exosomes containing miR-19b-3p [46]. Interleukin 1 receptor associated kinase 4 (IRAK4) plays a critical role in innate immune signaling by TLR, and loss of IRAK4 activity in mice and humans increases susceptibility to bacterial infections and causes defects in TLR and IL1 ligand sensing. Research shows a mechanism by which IRAK4 activity regulates TAK1 and IKKβ activation, leading to the induction of inflammatory cytokines in macrophages through NF-κB [47]. In addition to these molecules, some drugs can also induce the phenotypic transformation of macrophages. Losartan is a drug that blocks angiotensin receptors and induces macrophages to change from pro-inflammatory to anti-inflammatory type by regulating the NF-κB pathway [48].

## 6. Anti-Inflammation and Repair

**Figure 3:**
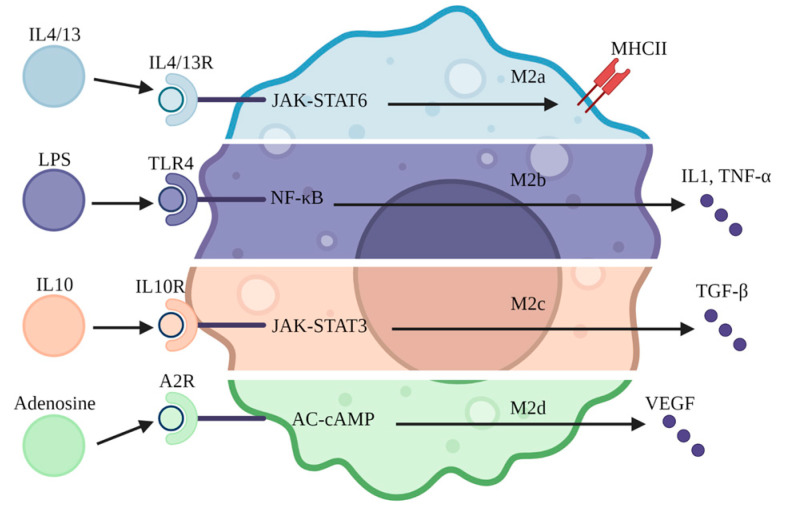
Different types of M2 macrophages. M2 macrophages can be divided into four types: M2a, stimulated by IL-4 and IL-13, can express more major histocompatibility complex II (MHCII) and has the function of antigen presentation; M2b, when stimulated by LPS, can express more IL-1β and TNF-α, which is more similar to pro-inflammatory macrophages; M2c, which is stimulated by IL-10 to produce more transforming growth factor beta (TGF-β) and is more consistent with the traditional view of anti-inflammatory macrophages; and M2d, which receives the action of adenosine, mainly produces vascular endothelial growth factor (VEGF) and may play a role in angiogenesis and circulation.

Macrophages exert their anti-inflammatory functions mainly by secreting cytokines and acting as antigen-presenting cells. The traditional view is that M2 macrophages are the main anti-inflammatory cells [49]. However, more and more studies have pointed out that M2 can be affected by different cytokines and play a variety of different functions. At present, M2 is divided into four types according to the different activating factors: M2a, M2b, M2C, and M2d [50]. M2a cells express more major histocompatibility complex II when activated by IL-4 and IL-13. Some scholars have suggested that this elevated expression may be the result of cell reprogramming because it is similar to the most primitive state of renal macrophages and can better activate T cells as antigen-presenting cells [12]. M2b cells are more similar to pro-inflammatory macrophages because they release pro-inflammatory cytokines and may play a larger role in helping to identify pathogens [51]. M2c cells are most similar to traditional anti-inflammatory macrophages because they can be activated by IL-10 to produce TGF-β [52], an important anti-inflammatory cytokine [53]. Macrophages regulate the proliferation of renal tubular epithelial cells through cytokines, such as IL-22, that contribute to the recovery of renal function [54]. The application of IL-22 can promote the regeneration and repair of renal tubular epithelial cells in SAKI [55]. It can also induce fibroblasts to secrete more collagen through TGF-β signaling [56], which may lead to renal fibrosis and a permanent and irreversible decline in renal function. M2d cells are mainly activated by extracellular adenosine. Through the production of cytokines such as vascular endothelial growth factor, M2d may play a greater role in regulating microcirculation (Figure 3) [57].

In addition to the interaction between macrophages and renal tubular epithelial cells, the association between macrophages and endothelial cells is equally important in influencing SAKI. In sepsis, endothelial cells express inflammatory cell-related adhesion molecules such as P-selectin, E-selectin, vascular cell adhesion molecule 1 (VCAM1), and intercellular adhesion molecule 1 (ICAM1). This allows circulating macrophages in the blood to be recruited to the kidneys. At the same time, macrophages can also release angiogenesis factors and regulate the function of endothelial cells. Recently, new studies have been produced on the effect of the interaction between macrophages and endothelial cells on SAKI [58]. Using a murine model of septic AKI, Privratsky et al. find that F4/80^hi^ kidney macrophages are necessary to attenuate the severity of septic AKI through elaboration of anti-inflammatory mediators, namely a receptor antagonist for IL-1 (also called IL1rn), which curbs IL-6 expression in endothelial cells. Using external datasets of murine endotoxemia and human COVID-19, other conditions associated with inflammatory kidney injury, they validated the high expression of IL1rn in macrophages and IL-6 in endothelial cells. The results provide further evidence of a macrophage–endothelial immunoregulatory axis where, within cortical regions of the kidney, macrophages and endothelial cells are primed to form a paracrine regulatory feedback loop to limit inflammatory injury in the kidney in the septic state [59]. These studies provide further rationale to target macrophage–endothelial interactions to protect the kidney during sepsis.

## 7. Targeting Macrophages to Treat SAKI

In view of the fact that macrophages play different roles in SAKI, regulating the function of macrophages may be important for the treatment of SAKI. Many substances targeting macrophages have been identified, such as mucin1 [60], resveratrol [61], quercetin [62], rhodomeroterpene [63], siRNA-TNF-α [64], naringin [65], and vitamin C [66]. These substances have been shown to block the binding of LPS and Toll-like receptor 4, reduce the inflammatory response of macrophages, and induce the transformation of macrophages from pro-inflammatory to anti-inflammatory. But it’s important to note that, some substances, such as Vitamin C, have not been shown to improve patient outcomes in clinical trials [67]. It is important to note that these treatments, either by blocking the binding effect between LPS and macrophage surface TLR4 or by inhibiting the phenotypic transformation of macrophages or their ability to produce inflammatory cytokines, may carry a considerable degree of risk, as they may lead to excessive immunosuppression and thus serious infection potential. In addition, macrophage-based nanoparticles have also been used in the treatment of SAKI [68]. The macrophages have the ability to cross physiological barriers and escape immune recognition and intracellular trafficking and have the ability to release potent pro-inflammatory cytokines, and therefore macrophage membrane coated nanoparticles have been exploited in the development of various therapeutics. Macrophage-derived nanoparticles or human functional synthetic nanoparticles can reduce tissue and cell damage [69], including antimicrobial peptides and cathepsin B [70], and plasmid DNA that can kill bacteria [71]. Therefore, nanomaterials have the potential to treat macrophage-associated diseases, especially sepsis. Except for treatment, numerous clinical monitoring technologies of nanoparticles are emerging, such as electrochemical and immunosensors for identifying infections, organ dysfunction, and immune dysregulation state. Detecting the localization of macrophages via nanomaterials can determine the severity of organ and tissue damage, thereby monitoring the progression of various macrophage-related diseases in real time. Although indirectly recognizing the pro-/anti-inflammatory cytokines’ lack of specificity, it provides directable roles in observing the inflammatory state of macrophage-associated diseases. Furthermore, finding sepsis-specific biomarkers remains a legacy challenge. There is no doubt that the introduction of nanotechnology into preclinical studies in sepsis associated macrophage therapeutics has made remarkable progress and has become a prospect for clinical applications [72]. However, there are currently no large clinical trials targeting macrophage therapy for SAKI. These are expected to be used in the treatment of SAKI patients in the future (Table 2).

## 8. Conclusions

The purpose of this paper was to explore the role of macrophage phenotype and phenotypic transformation in SAKI, which is of great significance for understanding the pathogenesis of SAKI and finding potentially effective therapeutic drugs. The surface markers of macrophages in different physiological states suggest that there may be many subtypes of macrophages, each of which may play a different role. Macrophages also exhibit phenotypic transformation across different stages of SAKI, playing pro-inflammatory, anti-inflammatory, and repair roles. These findings will be helpful for developing better treatment methods in the future.

## Figures and Tables

**Table 1:** Types of macrophages.

	Types					Role
Old	M1	CD11b, CD11c, CD68, CD86, F4/80 (EMR1 in human), Ly6C, MHCII, iNOS				Pro-inflammation
	M2	CD11b, CD11c, CD68, CD86, F4/80 (EMR1 in human), Ly6C, CD206, CD163				Anti-inflammation
New		CD11b	CD11c	F4/80	Other	
	1	high	high	low		Secreting IL-1β and TNF-α, activating Treg cells
	2	high	low	medium	Ly6C	The ability of phagocytosis is higher, but the ability to stimulate T cells is weak
	3	medium	medium	high	CX3CR1	Secreting IL-10, activating Th2 cells
	4	medium	high	low	CD103	The ability to activate Th1 cells is the strongest.
	5	low	medium	low		NA
		CD11b	CD11c	F4/80	CD206	
	1	high	high	low	A:+ B:-	The phagocytic ability of A is higher than B.
	2	high	low	medium	A:+ B:-	The phagocytic ability of A is higher than B.
	3	medium	medium	high	A:+ B:-	The phagocytic ability of A is higher than B.
	4	medium	high	low	A:+ B:-	The amount of A is lower than B during acute stage, and gradually decreases with time.
	5	low	medium	low	A:+ B:-	The amount of A is lower than B during acute stage, and gradually decreases with time.
		CD74 + CD81+				Common markers for mouse, rat, pig, and human renal macrophages

CD: a cluster of differentiation; EMR1: EGF-like module-containing mucin-like hormone receptor-like 1; MHCII: major histocompatibility complex class II; iNOS: inducible nitric oxide synthase; IL: interleukin; TNF: tumor necrosis factor; Treg cells: regulatory T cells; Th cells: T helper cells; CX3CR1: CX3C motif chemokine receptor 1.

**Table 2:** Treatment of macrophages.

Molecule	Introduction	Role
Mucin 1	Glycoproteins on the surface of epithelial cells	Blocking the binding of LPS and TLR4
Resveratrol	The natural phenol released by plants when attacked by pathogens	Blocking the binding of LPS and TLR4
Quercetin	A plant flavonol widely found in fruits and vegetables	Blocking the binding of LPS and TLR4
Rhodomeroterpene	New carotenoids isolated from rhododendron	Reducing the inflammatory response of macrophages
siRNA-TNF-α	A siRNA that can specifically knock out TNF-α	Reducing the inflammatory response of macrophages
Naringin	A flavonoid from citrus fruits, especially grapefruit	Inducing the transformation of macrophages from pro-inflammatory to anti-inflammatory
Vitamin C	A natural antioxidant is widely found in fruits and vegetables	Inducing the transformation of macrophages from pro-inflammatory to anti-inflammatory
Macrophage nanoparticles	Nanoparticles based on macrophages can absorb substances such as LPS.	Reducing tissue and cell damage
Antimicrobial peptides and cathepsin B (nanoparticles)	Nanoparticles formed on the basis of macrophages, having certain bactericidal activity.	Reducing tissue and cell damage
Plasmid DNA (nanoparticles)	Nanoparticles formed on the basis of macrophages, carrying antibacterial genes	Reducing tissue and cell damage

LPS: lipopolysaccharide; TLR4: toll-like receptors 4; siRNA: small interfering RNA; TNF-α: tumor necrosis factor alpha.
